# Vascular endothelial growth factor-A promoter polymorphisms, circulating VEGF-A and survival in acute coronary syndromes

**DOI:** 10.1371/journal.pone.0254206

**Published:** 2021-07-14

**Authors:** Barry R. Palmer, Melinda A. Paterson, Chris. M. Frampton, Anna P. Pilbrow, Lorraine Skelton, Chris J. Pemberton, Robert N. Doughty, Chris J. Ellis, Richard W. Troughton, A. Mark Richards, Vicky A. Cameron

**Affiliations:** 1 Department of Medicine, Christchurch Heart Institute, University of Otago Christchurch, Christchurch, New Zealand; 2 School of Health Sciences, College of Health, Massey University, Wellington, New Zealand; 3 Faculty of Medicine and Health Sciences, Department of Medicine, University of Auckland, Auckland, New Zealand; 4 Cardiovascular Research Institute, National University of Singapore, Singapore, Singapore; Medizinische Universitat Graz, AUSTRIA

## Abstract

**Background:**

Development of a competent collateral circulation in established coronary artery disease is cardio-protective. The vascular endothelial growth factor (VEGF) system plays a key role in this process. We investigated the prognostic performance of circulating VEGF-A and three genetic variants in the *VEGFA* gene in a clinical coronary cohort.

**Methods and results:**

The Coronary Disease Cohort Study (CDCS) recruited 2,140 patients, with a diagnosis of acute coronary syndrome (ACS), after admission to Christchurch or Auckland City Hospitals between July 2002 and January 2009. We present data for 1927 patients from the cohort genotyped for three SNPs in the VEGF-A gene, rs699947 (C-2578A), rs2010963 (C405G) and rs3025039 (C936T). Plasma VEGF-A concentrations were assayed in a subgroup (n = 550) of CDCS patients (geometric mean 36.6 [34.7–38.5] pg/ml). VEGF-A levels correlated with patient heart rate at baseline (p = 0.034). None of rs699947, rs3025039, nor rs2010963 genotypes were significantly associated with VEGF-A levels, but rs3025039 genotype was positively associated with collateral vessels perfusion according to the Rentrop classification (p = 0.01) and baseline natriuretic peptide levels (p<0.05). Survival in the CDCS cohort was independently associated with baseline VEGF-A levels and (in males) with rs699947 genotype.

**Conclusions:**

This study is strongly suggestive that VEGF-A levels have value as a prognostic biomarker in coronary heart disease patients and SNPs in *VEGF-A* deserve further investigation as prognostic markers and indicators of angiogenic potential influencing the formation of collateral circulation.

## Introduction

An important process ameliorating adverse outcomes after acute coronary occlusion is the formation of a functional collateral circulation around blocked arteries. Well-developed coronary collateral arteries are associated with improved survival in patients with coronary artery disease [1]. VEGF-A is a key factor in forming new blood vessels (angiogenesis) and collateral circulation (arteriogenesis), mediated by VEGF-A binding to the receptors VEGFR-1 (Flt-1) and VEGFR-2 (KDR) [2]. Polymorphisms in VEGF genes influence the expression of VEGF-A [3–5], and therefore possibly ability to form collateral circulation. A diverse set of single nucleotide polymorphisms (SNPs) at a variety of loci are associated with circulating VEGF-A concentrations [6]. *VEGFA* SNPs have been associated with CHD susceptibility [7,8]. VEGF-A is the prototype member of the VEGF family and was first cloned in 1989 [9]. Four other members of the human VEGF family have been identified: VEGF-B, VEGF-C (also called VEGF-2), VEGF-D, and placental growth factor (PlGF). VEGF-A is a homodimeric 34–42 kDa, heparin-binding glycoprotein with potent angiogenic, mitogenic and vascular permeability-enhancing activities specific for endothelial cells [2]. The well characterized heparin-binding isoform, VEGF165, is the principal effector of VEGF action [10].

VEGF-triggered downstream signaling in the vascular endothelium is mediated by three receptors, VEGFR-1, VEGFR-2, and VEGFR-3 (also known as Flt-4), belonging to the class III subfamily of receptor tyrosine kinases. VEGF-A binds to both VEGFR-1 and VEGFR-2. All three receptors contain seven immunoglobulin-like repeats in their extracellular domain and kinase insert domains in their intracellular region. They regulate VEGF family-mediated vasculogenesis, angiogenesis, and lymphangiogenesis and also mediate neurotrophic activity and regulate hematopoietic development. VEGFR-2 is thought to be the primary inducer of VEGF-mediated blood vessel growth, while VEGFR-3 plays a significant role in VEGF-C and VEGF-D-mediated lymphangiogenesis [11,12].

Anti-VEGF treatment as anti-angiogenic therapy in cancer is associated with an increased risk of adverse effects including MI, hypertension, proteinuria, arterial thromboembolism, cardiac ischemia and dysfunction affecting 1–15% of treated patients [13,14]. Conversely, VEGF-A might play a culprit role in atherogenesis and plaque instability via proinflammatory and angiogenic mechanisms [15,16]. However the contribution of VEGF to atherogenesis has been challenged. A *VEGFA* polymorphism associated with higher *VEGFA* expression was found to be associated with a lower risk of coronary artery disease in an epidemiological study [17]. Plasma levels of VEGF-A are increased in stable post-myocardial infarction (post-MI) patients compared to controls, correlating with inflammatory cytokines, but not atherosclerotic burden [18]. The sustained elevation in *VEGFA* expression during atherogenesis may be secondary, rather than causative, to inflammation and hypoxia in evolving lesions [2]. This study investigated the utility of three VEGF gene variants, previously shown to affect VEGF expression, and baseline plasma VEGF-A levels as prognostic markers in a well-characterized cohort of acute coronary syndromes patients (ACS) over prolonged follow up [19–22].

## Materials and methods

### Study population—Coronary disease cohort study

The Coronary Disease Cohort Study (CDCS) recruited 2140 patients, with a diagnosis of ACS, after admission to Christchurch or Auckland City Hospitals, New Zealand, between July 2002 and January 2009. Inclusion criteria included ischemic discomfort plus one or more of ECG change (ST-segment depression or elevation of ≥0.5 mm, T-wave inversion of ≥3 mm in ≥3 leads, or left bundle branch block), elevated levels of cardiac markers, a history of coronary disease, age of ≥65 years, and a history of diabetes or vascular disease [23]. Patients with serious co-morbidity (e.g. end-stage renal failure, cancer), that limited their life expectancy to <3 years, were excluded. Recruitment included a wide spectrum of age, both genders and significant sub-groups with established risk factors for CHD including hypertension and type II diabetes. Plasma for VEGF-A assay was collected at a baseline clinic, a median of 18 days after the index admission for ACS. We collected demographic and clinical data at baseline, including blood pressure, height, weight, ECG, echocardiography, family and personal medical history and medication regimes. Plasma samples were also assayed for natriuretic peptides and other analytes. Patients were followed for a median of 5.04 (0.11–9.49) years. Patients attended follow-up clinics 3–5 months and 12–14 months post-onset of ACS and participants completed questionnaires at 2 and 3 years post-discharge. Ethnicity was self-declared and categorized as Maori/Pacifika (Pacific Islander), European, Other or Unknown. Standardized transthoracic echocardiography was performed at baseline and at each follow-up clinic at Christchurch Hospital and University of Auckland clinics as described previously [19,24]. The study was approved by the New Zealand (NZ) Multi-Region Ethics Committee, retrospectively registered at the Australian New Zealand Clinical Trials Registry (ACTRN12605000431628 on 16 September 2005), and all participating patients provided written, informed consent.

### Clinical events

Clinical events were determined from recruitment questionnaires, planned follow-up clinic visits, consultation of patient notes, the NZ Ministry of Health and hospital Patient Management System databases, linked using the National Health Index number for each patient. Survival times were calculated from the date of index admission. The investigation conforms to the principles outlined in the Declaration of Helsinki and Title 45, U.S. Code of Federal Regulations, Part 46.

### Analyte measurements

Plasma samples were collected and stored in sealed tubes at -80°C as previously described [24]. VEGF-A was analyzed using a chemiluminescent quantitative sandwich enzyme immunoassay (R&D Systems, Inc. Minneapolis). Assay of baseline plasma samples for sFlt-1 (sVEGFR1) in this cohort has been described elsewhere [24]. Circulating levels of natriuretic peptides were assayed as previously described [25]. Interassay variation ranged from 3.3% (NT-proBNP) to 11.8% (BNP). Levels of VEGF-A at baseline were assayed in baseline plasma samples from 550 CDCS participants with the earliest recruitment dates in the cohort in order to maximize numbers of events on follow up for inclusion in survival analyses (recruited between July 2002 and August 2007).

### Evaluation of angiographic data

In the Christchurch subgroup of the cohort, collateral vessels were graded according to the Rentrop classification: 0: no filling of any collateral vessels, 1: filling of side branches of the artery to be perfused by collateral vessels without visualization of the epicardial segment; 2: partial filling of the epicardial artery by collateral vessels; and 3:complete filling of the epicardial artery by collateral vessels [26]. Those performing the grading were blinded to other clinical data. Coronary artery anatomy, severity of coronary stenoses and the myocardium at risk were assessed according to the Brandt score [27] in the same subgroup.

### DNA extraction and SNP genotyping

DNA samples were obtained for 2067 CDCS cohort patients. Extraction of genomic DNA for genotyping was performed as described previously [22,24]. DNA samples were genotyped for the rs699947 (C-2578A, assay ID C_8311602_10), rs2010963 (C405G, assay ID C_8311614_10) and rs3025039 (C936T, assay ID C_16198794_10) polymorphisms in *VEGFA* using 5 μL reaction volumes in 384-well plates with allele-specific TaqMan genotyping probes (ThermoFisher Scientific). Genotyping reactions including 1x Roche LightCycler 480 Probes Master mix and 100ng of genomic DNA were performed in a Roche LC480 (Roche Diagnostics Ltd., Rotkreuz, Switzerland) as described elsewhere [24]. Linkage disequilibrium data as determined for the 3 SNPs is shown in S1 Table. As quality control a random selection of 10% of samples were re-genotyped with 100% concordance with the original genotypes.

### Statistical analysis

Univariate analyses were performed to test for associations between SNP genotype and demographics, analyte levels and echocardiographic measurements using χ^2^ and ANOVA tests with Bonferroni correction. Skewed data were log-transformed before analysis and geometric means with 95% confidence intervals reported and adjusted for age, and the time between index admission and baseline sampling. The survival of stratified groups was compared using Kaplan-Meier analysis and the log-rank test. Independent associations between genotype and survival were tested using Cox proportional hazards multivariate analysis including the following established predictors: age [28,29], gender [30], previous MI [29], antecedent hypertension [31], β-blocker treatment [32], physical activity [33], and NT-proBNP levels [25]. Multivariate linear regression models were based on covariates showing univariate association with VEGF-A.

Assuming an overall mortality of 32% in CDCS, the study had 90% power to detect a hazard ratio (HR) >1.46 as statistically significant (two tailed α<0.05) for analysis of rs699947 genotypes (MAF = 0.491) and for rs3025039 (MAF = 0.140) >90% power to detect an odds ratio of 1.60. For patients assayed for VEGF-A there was 90% power to detect a difference of 10 pg/ml as statistically significant (two tailed α<0.05, assuming SD = 35). Ethnicity was self-declared and categorized as Maori/Pacific Islander, European, Other or Unknown, or in some analyses European versus Non-European. An additive genetic model was used unless stated otherwise. All analyses were performed using SPSS version 25 (IBM, Armonk, USA). There was no imputation of missing data with approximately 1% of cases missing measures utilised in the multivariable Cox regression analyses of the VEGF SNP genotypes. The baseline characteristics of the n = 550 cohort with VEGF-A plasma samples are compared with the remainder of the study group. Unadjusted p-values without correction for multiple testing are reported. Statistical significance was set at the 5% level (p<0.05).

## Results

### VEGF SNP genotypes and CDCS cohort data

Baseline characteristics of the CDCS cohort are summarized in Table 1. Proportions of self-declared Ethnicity were found to be Maori/Pacifika (6.3%), European (87.2%), Other (3.6%) or Unknown (2.9%) (see Table 1). Genotypes were obtained for 1927 patients from the CDCS cohort for all three SNPs, rs3025039 (C936T), rs2010963 (C405G) and rs699947 (C-2578A) in *VEGFA*. Genotype frequencies were rs3025039: CC, 73.9%; CT, 24.2% and TT, 1.9% (minor allele frequency [MAF] = 0.140); rs2010963 GG, 46.7%; GC 43.6% and CC 9.7% (MAF = 0.315); rs699947 CC 26.2%; CA 49.4% and AA 24.4% (MAF = 0.491) and all genotypes conformed to the Hardy-Weinberg equilibrium (p≥0.769). MAF did not differ between ethnic groups (p≥0.280). Baseline patient characteristics for each genotype group are shown in Table 2 (rs699947) and S3 & S4 Tables (rs3025039 and rs2010963). Patients with genotype rs699947 AA were physically active less frequently than other genotype groups (Table 2). Scoring of angiograms from patients in the cohort for collateral circulation using the Rentrop grading system revealed that rs3025039 was significantly associated with Rentrop score (n = 587 with data for both), with patients with TT genotype having higher scores than the CC or CT genotype groups (p = 0.032) (S2 Table). No significant associations with baseline characteristics and other SNP genotypes were observed.

**Table 1:** Baseline characteristics of the CDCS cohort.

Baseline characteristics	n	Mean ± SE or n (%)
Male Gender	1927	1378 (71.5%)
Index event diagnosis: Unstable Angina	1927	516 (26.8%)
ST-elevation MI	1927	427 (22.2%)
Non-ST-elevation MI	1927	984 (51.1%)
Age at baseline (years)	1927	66.7 ± 0.28
Ethnicity (European, Maori & Pasifika, Other, Unknown)	1927	87.2%,6.3%,3.6%,2.9%
Previous MI	1909	569 (29.5%)
Previous Heart Failure	1914	183 (9.5%)
Antecedent Hypertension	1907	999 (51.8%)
Type II diabetes	1918	312 (16.2%)
Renal disease	1908	190 (9.9%)
BMI (kg/m^2^)	1907	27.4 ± 0.11
Tobacco Use (never smoked)	1923	703 (36.5%)
Alcohol Use (non-drinker)	1921	494 (25.6%)
LVEF	1857	57.4%±0.28
***Discharge Medications***		
ACE inhibitor	1927	1098 (57.0%)
β-blocker	1927	1680 (87.2%)
Diuretic	1927	528 (27.4%)
Statin	1927	1706 (88.5%)
Dual antiplatelet therapy	1927	1006 (52.3%)

**Table 2:** Baseline characteristics, drug treatment and neurohormonal data for CDCS patients stratified by VEGF SNP genotype group.

*VEGFA* C-2578A rs699947 Genotype							
	n	CC	n	CA	*n*	AA	*p*
Age (years)$	50530	66.0±0.55	949	66.8±0.40	469	67.2±0.57	0.257
Male Gender	505	349 (69.1%)	951	684 (71.9%)	471	342 (72.6%)	0.411
BMI (kg/m^2^) $	495	27.6±0.24	938	27.3±0.15	463	27.9±0.24	0.138
Physical Activity‡	466	1,17.8%; 2, 14.2%; 3, 11.8%; 4,56.2%; 3; 3	886	1,22.0%; 2,10.3%; 3,13.5%; 4,54.2%	436	1,22.7%; 2,13.8%; 3,16.7%; 4, 46.8%	0.010
LVEF	492	57.7±0.57	914	57.4±0.39	451	57.1±0.60	0.743
**History**							
Previous Myocardial Infarction$	502	143 (28.5%)	943	271 (28.7%)	464	155 (33.4%)	0.149
Hypertension $	502	263 (52.4%)	942	485 (51.5%)	463	251 (54.2%)	0.630
Diabetes$	504	82 (16.3%)	948	147 (15.5%)	466	83 (17.8%)	0.544
Renal Disease$	501	49 (9.8%)	943	92 (9.8%)	464	49 (10.6%)	0.883
Alcohol (Non-Drinkers)$	505	123 (24.4%)	951	257 (27.0%)	471	114 (24.2%)	0.002
**Analytes**							
Plasma Creatinine$$ (mmol/l)	490	94.1 (91.7–96.5)	919	94.1 (92.5–95.7)	457	94.5 (92.4–96.8)	0.322
BNP (pmol/L) $$	501	16.2 (15.0–17.6)	941	16.8 (15.9–17.9)	468	18.4 (17.0–20.0)	0.080
NT-proBNP (pmol/L) $$	501	73.8 (67.2–81.0)	941	74.7 (69.6–80.0)	468	83.9 (76.0–92.6)	0.108
sFlt-1 (pg/mL)	121	109 (101–118)	236	105 (99.1–111)	136	106 (98.1–115)	0.710
VEGF-A (pg/mL)	131	37.8(33.6–42.6)	264	36.7 (34.1–39.5)	155	35.4 (32.2–38.8)	0.658
**Discharge Medications**							
ACE inhibitor $	505	301 (59.6%)	949	541 (57.0%)	469	254 (54.2%)	0.230
β-blocker $	505	443 (87.7%)	949	835 (88.0%)	469	399 (85.1%)	0.279
Diuretic$	505	146 (28.9%)	949	254 (26.8%)	469	127 (27.1%)	0.672
Statin$	505	452 (89.5%)	949	839 (88.4%)	469	412 (87.8%)	0.704
Amiodarone	505	21 (4.2%)	952	51 (5.4%)	471	24 (5.1%)	0.530
Clopidogrel	505	278 (55.0%)	952	511 (53.7%)	472	231 (49.0%)	0.190
***Angiographic measures***							
Rentrop score	140	0.51±0.07	315	0.47±0.04	131	0.53±0.07	0.310
Brandt score	264	3.31±0.19	526	3.37±0.14	231	3.32±0.21	0.821
Vessel Disease	264	2.09±0.06	529	1.99±0.04	233	2.09±0.06	0.305
Median Follow-Up (years) § (years)§	505	5.13 (0.14–9.48)	949	5.23 (0.11–9.49)	469	5.19 (0.14–9.49)	

^$^Means (SEM) or occurrence (percentage);

^$$^Geometric mean (95% confidence interval);

^§^Median (range).

^‡^Score of 1 = sedentary, 2 = <30 minutes activity on >2 days/week, 3 = ≥30 minutes on 2 days/week, 4 = ≥30 minutes on ≥3 days/week.

### *VEGFA* SNP genotypes and analyte measurements

Baseline plasma samples from 550 CDCS patients were assayed for VEGF-A (geometric mean 36.8±1.46 pg/ml). Baseline characteristics of this group are summarized and compared to the remainder of the cohort in S2 Table, some patient characteristics differed (p<0.05) between these groups including age and ethnicity. Patients with above-median VEGF-A levels were older (mean age: above median 70.5±0.94, below median 67.5±1.04 years, p = 0.038), had greater waist measurements (mean: above median 95.9±1.08, below median 92.0±1.00 cm, p = 0.008) and trended towards greater BMI (mean: above median 27.8±0.40, below median 26.8±0.38, p = 0.059). VEGF-A levels were weakly correlated with patient heart rate at baseline (n = 512, r = 0.094, p = 0.034). None of the genotypes for rs699947, rs3025039 or rs2010963 were significantly associated with VEGF-A levels. Baseline BNP (n = 1894; CC 17.4±1.02, CT 16.7±1.04, TT 13.2±1.13 pmol/L, p = 0.012) and NT-proBNP levels (n = 1908; CC 77.3±1.03, CT 75.4±1.05; TT 60.7±1.18 pmol/L, p = 0.038) adjusted for age and time to plasma sampling, were significantly associated with rs3025039 genotype.

### Clinical outcome in the CDCS cohort

**Fig 1:**
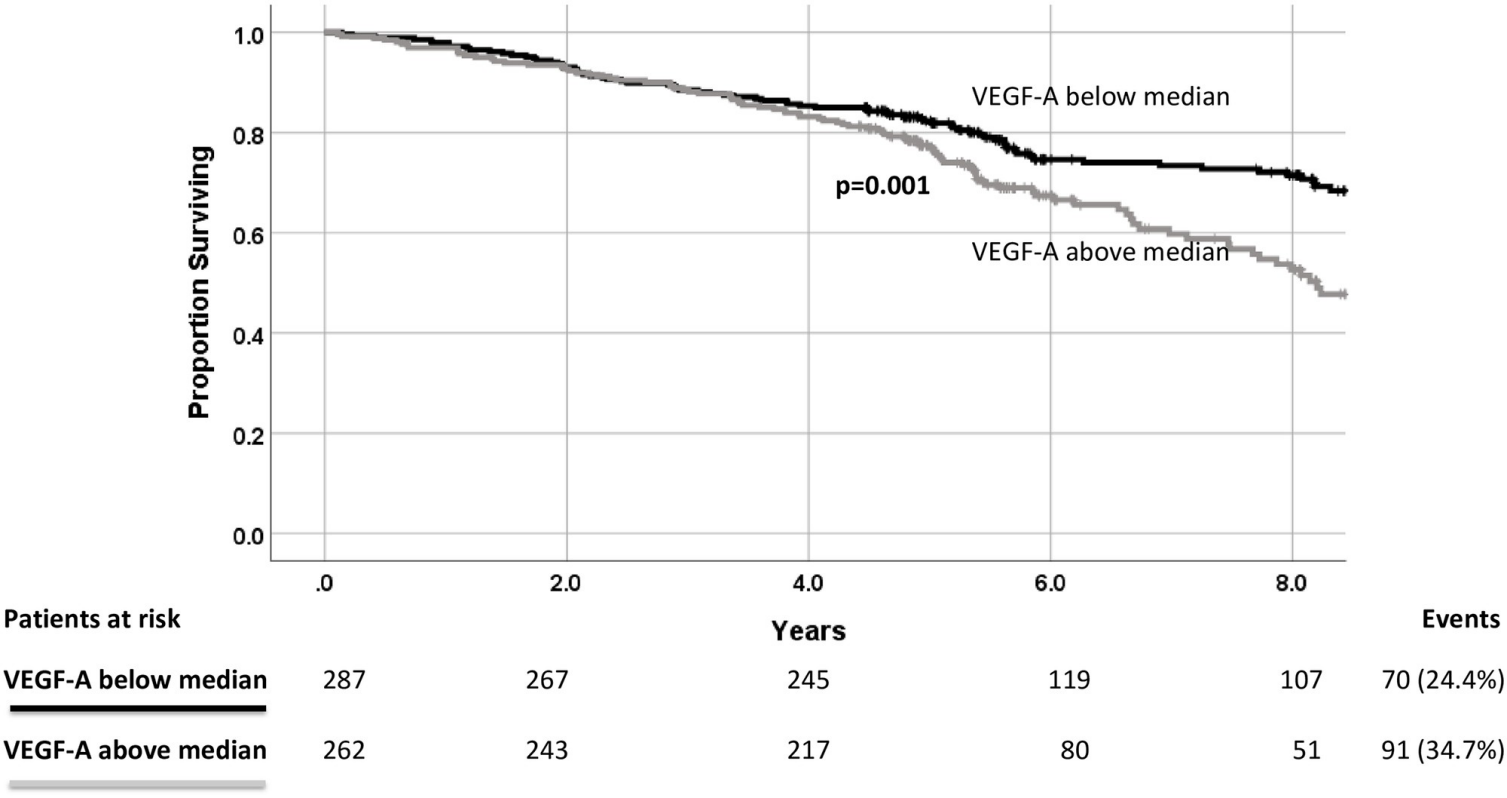
Kaplan-Meier survival plot for survival versus all-cause death in the CDCS cohort stratified by above and below median baseline VEGF-A levels.

**Fig 2:**
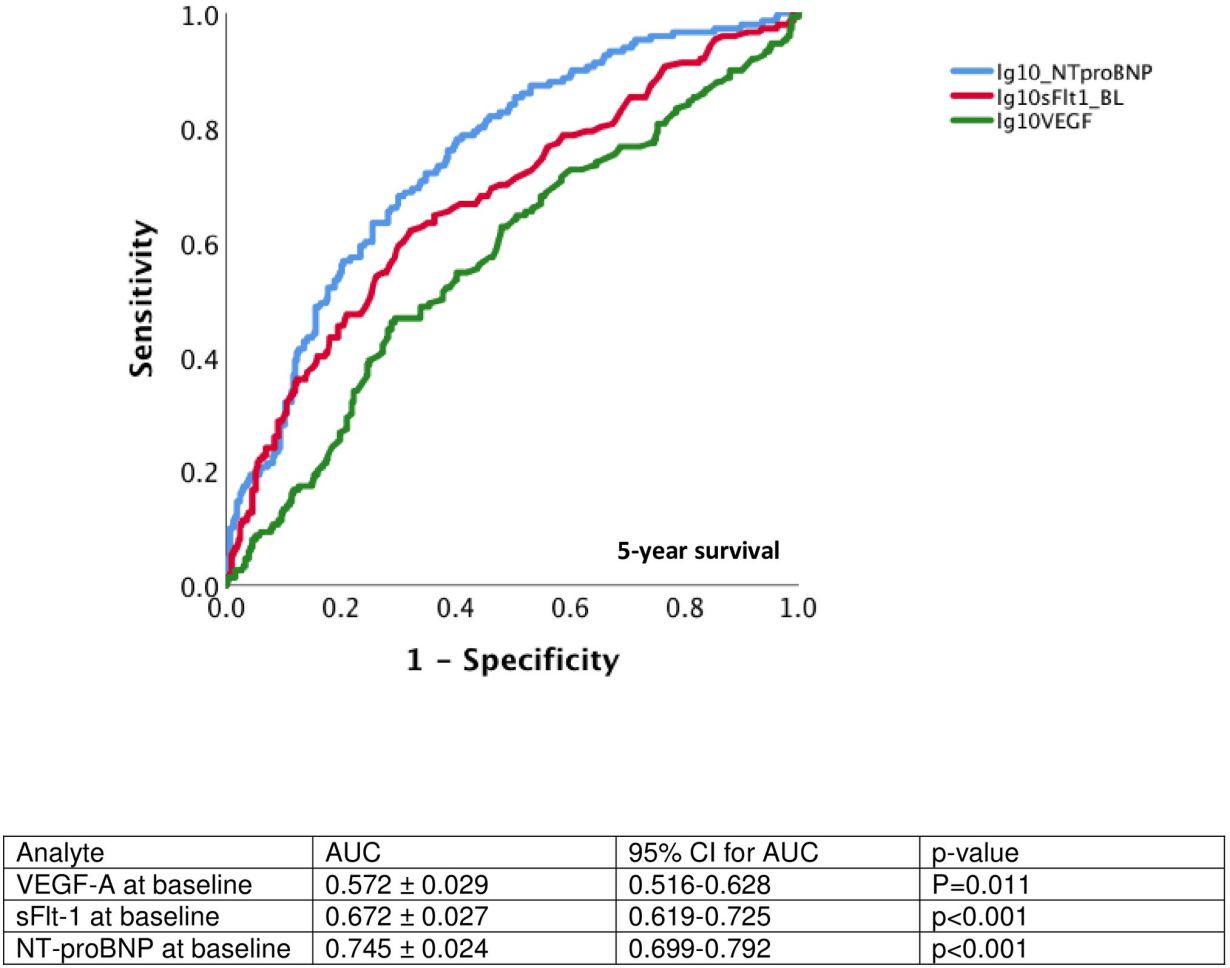
Receiver-operator curve analysis of VEGF-A, sFlt-1 and NT-proBNP as predictors of survival at 5 years in the CDCS cohort.

Patients with above-median VEGF-A levels were more likely to die during the follow-up period (all-cause mortality: above median 34.7%, below median 24.4%, p<0.001, events = 161) (Fig 1). High VEGF-A levels were associated with higher mortality due to cardiovascular causes (CVD mortality: above median 36.6%, below median 16.7%, p<0.001, events = 70). The subgroup of the cohort with both high VEGF-A and NT-proBNP levels had the greatest mortality, but only just significantly more than those with above median NT-proBNP, but low VEGF-A (p = 0.04) (S1 Fig). VEGF-A level at baseline was also an independent predictor of mortality in Cox proportional hazards models, both with a minimal set of covariates (Table 3), including NT-proBNP and sFlt-1, and the same covariates as used to assess rs699947 as a predictor. ROC analysis showed that baseline plasma VEGF-A was a poorer predictor of survival at 5 years when compared to NT-proBNP and sFtl-1 (Fig 2).

**Table 3:** Cox’s proportional hazards regression model including baseline VEGF-A and sFlt-1 levels for mortality in the CDCS cohort (n = 458, 139 deaths).

	Coefficient	SE	Wald	Significance	Hazard Ratio	95% CI for HR	
						Lower	Upper
Age at index admission	0.04	0.01	10.7	0.001	1.04	1.02	1.07
Log_10_ NT-proBNP at baseline^$^	0.73	0.27	7.45	0.006	2.08	1.23	3.51
Log_10_ VEGF-A at baseline^$^	0.77	0.30	6.62	0.010	2.16	1.20	3.89
Log_10_ sFlt-1 at baseline^$^	1.13	0.47	5.85	0.016	3.10	1.24	7.74
Physical Activity (scale 1–4)^$$^	-0.35	0.07	24.8	<0.001	0.70	0.61	0.81
Previous Myocardial Infarction	0.54	0.18	8.66	0.003	1.71	1.20	2.44
Atrial Fibrillation at baseline	0.22	0.11	4.03	0.045	1.24	1.01	1.54
Cockcroft-Gault—eGFR	-0.01	0.004	11.6	0.001	0.99	0.98	0.99

^$^Hazard Ratio represents the change in risk for every 10-fold increase in analyte level.

^$$^Score of 1 = sedentary, 2 = <30 minutes activity on >2 days/week, 3 = ≥30 minutes on 2 days/week, 4 = ≥30 minutes on ≥3 days/week.

Abbreviations: CI = confidence interval, eGFR = estimated glomerular filtration rate, HR = hazard ratio, MI = myocardial infarction, NT-proBNP = amino-terminal pro-b-type natriuretic peptide, sFlt-1 = soluble fms-like tyrosine kinase-1.

**Fig 3:**
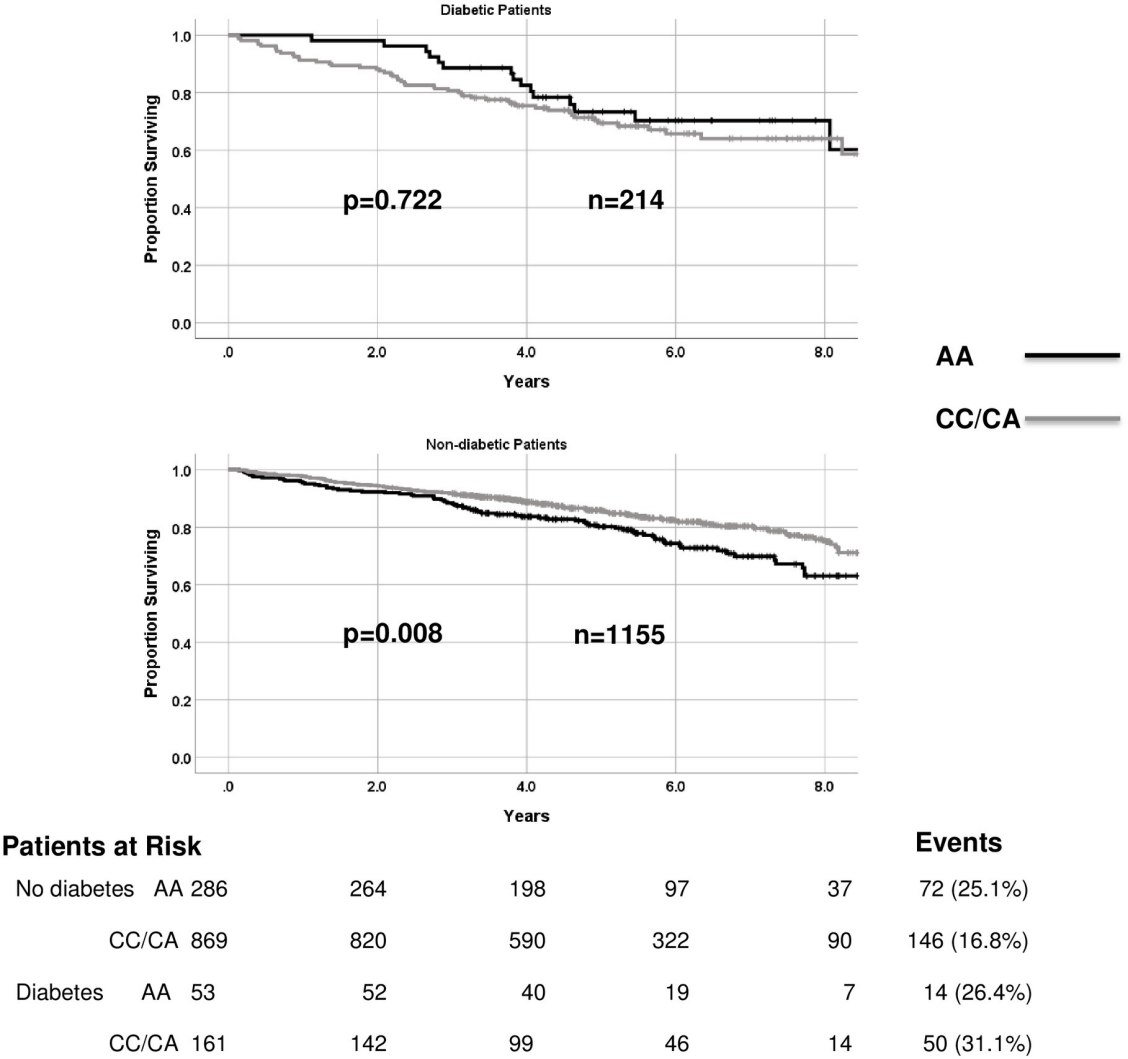
Kaplan-Meier survival versus all-cause death in male patients from the CDCS cohort for a) non-diabetic patients and b) type II diabetic patients stratified by rs699947 genotype CC/CA v AA.

Survival in the male subgroup of the CDCS cohort was significantly associated with rs699947 genotype in a multivariate Cox proportional hazards model of survival including rs699947 genotype and diabetic status (Table 4). This analysis revealed that the association of rs699947 genotype with mortality in males was dependent on an interaction with diabetic status. Mortality was associated with genotype (p = 0.008) in male patients without type II diabetes, but not in those with diabetes (in whom mortality was higher overall than in non-diabetic participants) (p = 0.722) (Fig 3). Survival in the female patients from the CDCS cohort was not associated with rs699947 genotype (p = 0.731), even when stratified by diabetic status (p>0.176). No other significant associations between SNPs and clinical outcomes were observed.

**Table 4:** Cox’s proportional hazards regression model for mortality in male patients from the CDCS cohort (n = 1215, 249 deaths).

	Coefficient	SE	Wald	Significance	Hazard Ratio	95% CI for HR	
						Lower	Upper
Age at index admission	0.06	0.01	46.4	<0.001	1.06	1.05	1.08
Log_10_ NT-proBNP at baseline ^$^	1.35	0.22	39.1	<0.001	3.86	2.3	5.90
ACE inhibitor at discharge	0.15	0.15	0.01	0.920	0.99	0.74	1.32
Amiodarone treatment at discharge	0.69	0.30	5.33	0.021	2.00	1.11	3.61
β-blocker treatment at discharge	0.30	0.21	2.02	0.155	1.35	0.89	2.03
Statin treatment at discharge	0.13	0.19	0.46	0.499	1.13	0.79	1.63
Physical Activity (scale 1–4) ^$$^	-0.17	0.06	8.86	0.003	0.84	0.75	0.94
Previous Myocardial Infarction	0.46	0.15	10.0	0.002	1.58	1.19	2.11
Previous Heart Failure	0.25	0.18	1.88	0.170	1.29	0.90	1.85
Antecedent Hypertension	0.14	0.14	1.07	0.302	1.15	0.88	1.51
Previous Cancer	0.07	0.17	0.16	0.694	1.07	0.77	1.49
Previous renal disease	0.55	0.17	10.4	0.001	1.73	1.24	2.42
Previous pulmonary disease	0.39	0.16	5.65	0.017	1.47	1.07	2.02
Atrial fibrillation	0.45	0.16	7.55	0.006	1.57	1.14	2.16
LVEF	0.00	0.01	0.00	0.986	1.00	0.99	1.01
NYHA score			9.58	0.023			
I v II	0.37	0.18	4.17	0.041	0.69	0.49	0.99
I v III	0.15	0.19	0.63	0.428	1.16	0.80	1.68
I v IV	0.24	0.21	1.30	0.25	1.27	0.84	1.90
BMI	0.004	0.02	0.04	0.845	1.00	0.97	1.04
Time to sampling	0.01	0.01	3.19	0.074	1.01	1.00	1.02
Ethnicity European v non-European	-0.26	0.26	1.02	0.313	0.77	0.46	1.28
Type II Diabetes	0.44	0.18	5.91	0.015	1.56	1.09	2.22
rs699947 genotype CC/CA v AA	0.80	0.34	5.60	0.018	2.23	1.15	4.35
Type II Diabetes x rs699947 genotype	0.92	0.37	6.22	0.013	2.52	1.22	5.20

^$^Hazard Ratio represents the change in risk for every 10-fold increase in BNP level.

^$$^Score of 1 = sedentary, 2 = <30 minutes activity on >2 days/week, 3 = ≥30 minutes on 2 days/week, 4 = ≥30 minutes on ≥3 days/week.

Abbreviations: BMI = body mass index, CI = confidence interval, HR = hazard ratio, LVEF = left ventricular ejection fraction, NT-proBNP = amino-terminal pro-b-type natriuretic peptide, NYHA = New York Heart Association.

## Discussion

The characterization of genetic polymorphisms of the *VEGFA* gene that influence the gene’s expression [3,4] and the central role of VEGF-A in blood vessel formation have made the gene an object of interest in studies of susceptibility to, and progression of, cardiovascular disease [34,35]. We found one of three SNPs assayed, rs699947, was an independent predictor of mortality in male non-diabetic participants in the CDCS cohort. Allele frequencies for all three SNPs genotyped in this study agreed closely with those reported for the CEU and GBR populations [36]. Reports of gender-specific expression of human VEGF-A are available [37], but not to our knowledge rs699947 x gender-specific associations with heart pathophysiology. VEGF-A release and angiogenesis has been shown to be stimulated by estradiol in human mesenchymal stem cells, that have the potential to differentiate into myocytes [38]. There is at least one other report of a SNP associated with VEGF-A expression interacting with metabolic syndrome status [39]. There are some reports that VEGF-A expression may be altered in diabetes [40], and diabetes and SNP genotype appear to interact to influence angiogenesis [41,42], which would fit with our observations. It would seem that the influence of rs699947 in normal VEGF angiogenic function is deranged in diabetes, and potentially more so in males than females, leading to greater plaque formation and instability. This hypothesis requires testing in more detailed studies.

The mean levels of VEGF-A we measured in our cohort were very similar or slightly elevated compared to those reported in healthy controls [43,44]. Above median levels of VEGF-A were found to be associated with mortality. This is in agreement with others [45]. While it has been hypothesized that the VEGF pathway is protective through its promotion of angiogenesis, and therefore countering the ischemic effects of coronary atherosclerosis, evidence exists that excess VEGF expression contributes to atherosclerotic plaque formation [2,16]. This suggests high levels of VEGF-A may be associated with elevated risk, perhaps by the mechanism of exacerbating plaque instability. Alternatively, high VEGF-A levels may represent a restorative, but inadequate, response to tissue ischemia from myocardial tissue with inadequate perfusion. There are reports of correlation of levels of VEGF-A and inflammatory markers [18], suggesting similar phenotypic profiles for effectors of the VEGF and inflammatory systems in patients with post-acute CHD. Mortality due to non-cardiovascular causes did not appear to be elevated in the subgroup of the cohort with high VEGF-A levels, as might be expected due to the role of VEGF-A in tumour angiogenesis [46]. The findings that high levels of VEGF-A are associated with increased mortality in this cohort are consistent with rs699947 having been previously implicated in the pathogenesis of CHD [47] and being a predictor independent of NT-proBNP and sFlt-1 suggests a complex regulatory network, although natriuretic peptides and VEGFA may respond to similar signalling is suggested by S1 Fig.

We also found that rs3025039 was significantly associated with Rentrop score, a measure of collateral vessel development. While the rs3025039 TT genotype group had higher levels of VEGF-A, but not significantly so, as others have found [48,49]. Other reports have also suggested a link between VEGF gene variants and angiographic measurements in CVD [50,51]. Those studies focused on stent restenosis and carotid artery stenosis, whereas our measurements examined the extent of coronary vessel disease and the degree to which collateral vessels were perfused. The polymorphisms that had significant clinical association (rs3025039 and rs699947) may be altering expression of VEGF-A at the cardiac tissue level, but significant SNP-associated differences were not detectable in circulating levels in this cohort due to factors associated with differential response to the coronary event and varying medication regimes.

Limitations of the study include: 1) Missing data for some parameters limited the power of this study to explore their association with genotype and VEGF levels; 2) The study cohort was dominated by ethnic Europeans and the results cannot be extrapolated to other populations with any certainty; 3) Blood samples were collected at varying times after the index event in order to avoid major influences from the acute event on plasma analytes, while this variable may have affected levels of these analytes, adjustment for time to sampling was included in statistical analysis in an effort to mitigate this; 4) The assay for VEGF-A was only conducted on a minority of the total CDCS cohort, which differed in age and ethnic profile from the remainder of the cohort, these covariates were included in multivariate analyses to correct for this; 5) The CDCS cohort was recruited over 10 years ago and therefore limited availability of recent treatment regimes, such as dual anti-platelet therapy (received by 54% of the CDCS cohort) may have affected clinical outcome endpoints.

### Conclusion

In summary, we report associations between plasma levels of VEGF-A, some of its genetic variants, patient characteristics and outcome measures. This, along with our recent report of similar findings with levels of sFlt-1, the soluble version of the VEGFR1 receptor [24], indicates that both plasma analytes and genetic markers from the VEGF system may have prognostic value in patients with cardiovascular disease. These observations suggest further investigation in larger, more diverse cohorts would be of value.

## Supporting information

S1 Fig Kaplan-Meier survival plot for survival versus all-cause death in the CDCS cohort stratified by groups formed by the combination of median splits for baseline VEGF-A and NT-proBNP levels. (TIFF)

S1 Table Linkage disequilibrium data (R^2^ data) for SNPs genotyped in this study. (PDF)

S2 Table Baseline characteristics of the CDCS cohort stratified by whether patient samples were assayed for VEGF-A or not. (PDF)

S3 Table CDCS cohort patient characteristics stratified by rs3025039 genotype. (PDF)

S4 Table CDCS cohort patient characteristics stratified by rs2010963 genotype. (PDF)
