# Supplementary figures and images for: Vascular endothelial growth factor-A promoter polymorphisms, circulating VEGF-A and survival in acute coronary syndromes
Source: PLoS One. 2021 Jul 14;16(7):e0254206. doi: 10.1371/journal.pone.0254206 (PMC8279389; doi:10.1371/journal.pone.0254206)

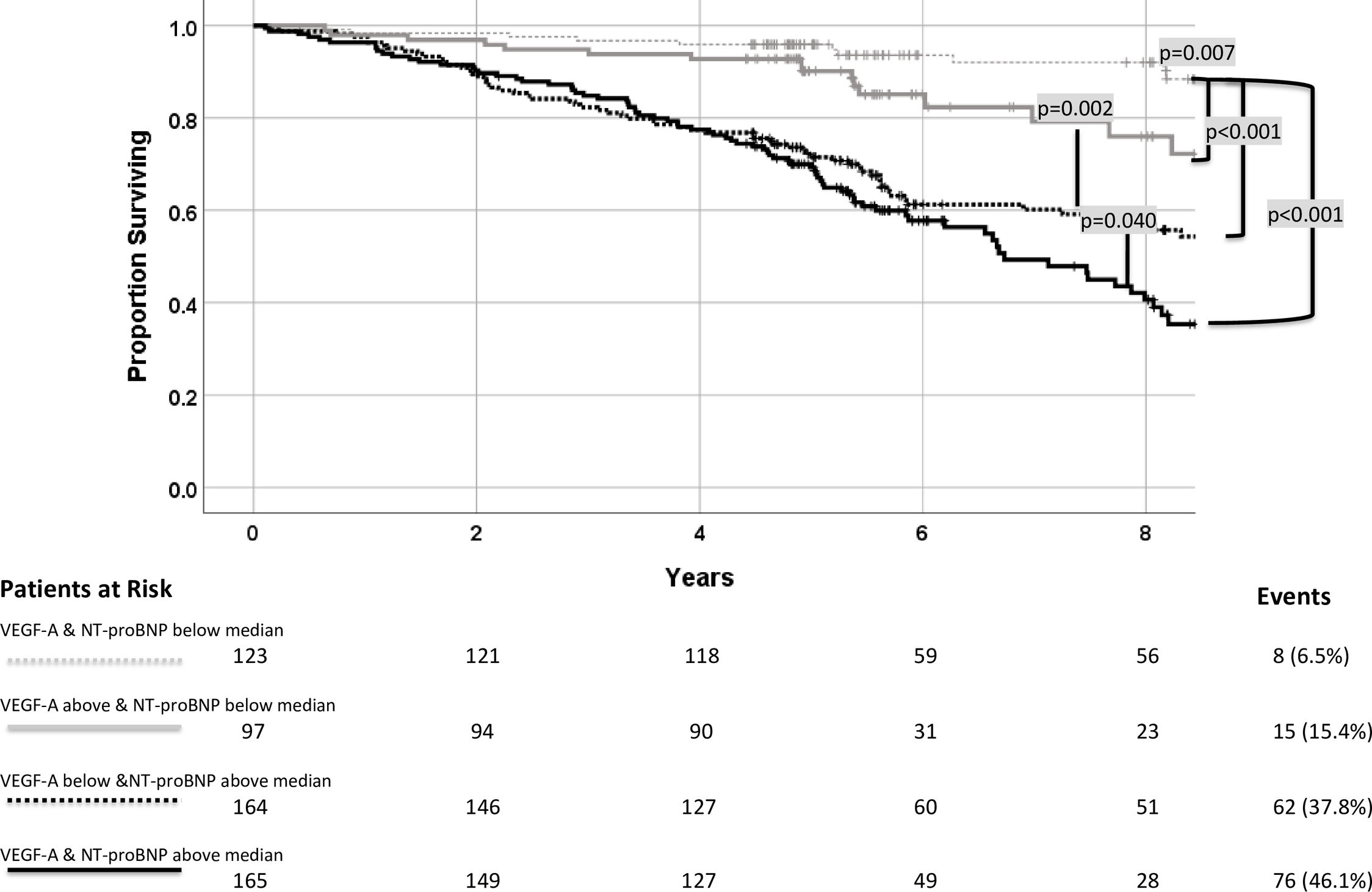

Supplement: S1 Fig — (TIFF) [file pone.0254206.s001.tiff]
