# Supplementary material for: Vascular endothelial growth factor-A promoter polymorphisms, circulating VEGF-A and survival in acute coronary syndromes
Source: PLoS One. 2021 Jul 14;16(7):e0254206. doi: 10.1371/journal.pone.0254206 (PMC8279389; doi:10.1371/journal.pone.0254206)
Supplement: S1 Table — (PDF) [file pone.0254206.s002.pdf]

S1 Table. Linkage disequilibrium data (R^2 data) for SNPs genotyped in this study.

rs_number	rs699947	rs2010963
rs699947	1.0	
rs2010963	0.468	1.0
rs3025039	0.0	0.002
