# Supplementary material for: Vascular endothelial growth factor-A promoter polymorphisms, circulating VEGF-A and survival in acute coronary syndromes
Source: PLoS One. 2021 Jul 14;16(7):e0254206. doi: 10.1371/journal.pone.0254206 (PMC8279389; doi:10.1371/journal.pone.0254206)
Supplement: S2 Table — (PDF) [file pone.0254206.s003.pdf]

S2 Table. Baseline characteristics of the CDCS cohort stratified by whether patient samples were assayed for VEGF-A or not.

Baseline characteristics	n	Mean ± SE or n (%)<br>Assayed for VEGF-A		n	Mean ± SE or n (%)<br>Not assayed for VEGF-A		p-value
Male Gender	550	383 (69.6%)		1377	993 (72.1%)		0.530
Age at baseline (years)	550	68.8±0.51		1377	65.9±0.33		<0.001
Ethnicity	550	European	512 (93.0%)	1377	European	1169 (84.9%)	<0.001
		Maori/Pasifika	20 (3.65)		Maori/Pasifika	102 (7.4%)	
		Other	5 (0.95)		Other	63 (4.6%)	
		Unknown	13 (2.45)		Unknown	43 (3.1%)	
Discharge Medications							
ACE inhibitor	550	302 (54.9%)		1377	797 (57.9%)		0.473
Amiodarone	550	28 (5.1%)		1377	68 (4.9%)		0.978
β-blocker	550	477 (86.7%)		1377	1203 (87.4%)		0.905
Clopidogrel	550	259 (47.1%)		1377	761 (55.3%)		0.006
Diuretic	550	167 (30.4%)		1377	362 (26.3%)		0.171
Statin	550	471 (85.6%)		1377	1235 (89.7%)		0.036
