# Supplementary material for: Vascular endothelial growth factor-A promoter polymorphisms, circulating VEGF-A and survival in acute coronary syndromes
Source: PLoS One. 2021 Jul 14;16(7):e0254206. doi: 10.1371/journal.pone.0254206 (PMC8279389; doi:10.1371/journal.pone.0254206)
Supplement: S3 Table — (PDF) [file pone.0254206.s004.pdf]

S3 Table. CDCS cohort patient characteristics stratified by rs3025039 genotype.

	VEGF-A C936T rs3025039 Genotype						
	n	CC	n	CT	n	TT	*p*
Age (years)$	1422	66.5±0.33	465	67.0±0.56	36	69.6±1.79	0.270
Male Gender	1424	1021 (71.4%)	467	330 (70.7%)	36	24(66.7%)	0.727
BMI (kg/m^2) $	1400	27.5±0.14	461	27.5±0.22	35	25.8±0.72	0.212
Physical Activity‡	1334	1,21.3%; 2,11.7%; 3,13.6%; 4, 53.4%	421	1,21.1%; 2,13.3%; 3,15.2%;4,50.4%	33	1,12.1%; 2,15.2%; 3,9.1%; 4,63.6%	0.583
LVEF	1373	57.2±0.33	450	57.9±0.57	34	60.9±1.54	0.430
History							
Previous MI$	1414	431 (30.5%)	459	133 (29.0%)	36	5 (13.9%)	0.090
Hypertension $	1411	741 (52.2%)	460	241 (52.4%)	36	17 (47.2%)	0.821
Diabetes$	1419	226 (15.9%)	463	82 (17.7%)	36	4 (11.1%)	0.465
Renal Disease$	1410	139 ((9.9%)	462	47 (10.2%)	36	4 (11.1%)	0.955
Alcohol (Non-Drinkers)$	1424	375 (26.3%)	467	112 (24.0%)	36	7 (19.4%)	0.837
Plasma Creatinine	1378	94.1 (92.8-95.5)	453	94.1 (91.9-96.4)	35	98.9 (88.8-110)	0.801
BNP (pmol/l) $$	1415	17.3 (16.5-18.2)	460	16.5 (15.2-18.0)	35	13.2 (10.2-17.1)	0.158
NT-proBNP (pmol/l) $$	1415	77.4 (73.2-81.9)	460	75.4 (68.1-83.5)	35	60.7 (43.7-84.5)	0.400
sFlt-1 (pg/mL)	374	108 (103-113)	111	102 (93.6-110)	8	99.5 (75.5-131)	0.427
VEGF-A (pg/mL)	413	36.1(33.9-38.4)	128	37.7 (34.2-41.6)	8	45.4 (22.6-91.2)	0.481
Discharge Medications							
ACE inhibitor $	1422	818 (57.5%)	465	262 (56.3%)	36	16 (44.4%)	0.400
β-blocker $	1422	1244 (87.4%)	465	405 (87.1%)	36	28 (77.8%)	0.347
Diuretic$	1422	401 (28.1 %)	465	119 (25.5%)	36	7 (19.4%)	0.427
Statin$	1422	1263 (88.8%)	465	408 (87.7%)	36	32 (88.9%)	0.757
Clopidogrel	1422	736 (51.7%)	465	260 (55.9%)	36	22(61.1%)	0.298
Amiodarone	1422	68 (4.8%)	465	25 (5.4%)	36	3 (8.3%)	0.625
Angiographic measures							
Rentrop score	426	0.51±0.04	147	0.39±0.06	14	1.00±23	0.032
Brandt score	763	3.27±0.11	243	3.46±0.20	22	4.24±0.70	0.424
Vessel Disease	761	2.00±0.03	242	2.08±0.06	23	2.17±0.17	0.511
Median Follow-Up$^{\$\$\$}$	1422	5.18 (0.11-9.49)	465	4.89 (0.14-9.48)	36	5.03 (3.18-9.47)	

$Means (SEM) or occurrence (percentage); $$Geometric mean (95% confidence interval) & adjusted for age and time to plasma sampling; $^{\$\$\$}$Median (range).

‡Score of 1=sedentary, 2=<30 minutes activity on >2 days/week, 3=≥30 minutes on 2 days/week, 4= ≥30 minutes on ≥3 days/week.
