# Supplementary material for: Vascular endothelial growth factor-A promoter polymorphisms, circulating VEGF-A and survival in acute coronary syndromes
Source: PLoS One. 2021 Jul 14;16(7):e0254206. doi: 10.1371/journal.pone.0254206 (PMC8279389; doi:10.1371/journal.pone.0254206)
Supplement: S4 Table — (PDF) [file pone.0254206.s005.pdf]

S4 Table CDCS cohort patient characteristics stratified by rs2010963 genotype.

	VEGF-A C405G rs2010963 Genotype						
	n	GG	n	GC	n	CC	*p*
Age (years)$	896	66.6±0.42	840	66.7±0.42	187	67.0±0.88	0.907
Male Gender (F/M)	896	648 (72.3%)	840	594(70.7%)	187	131(70.1%)	0.712
BMI (kg/m²) $	883	27.7±0.17	827	27.4±0.17	186	27.2±0.36	0.391
Physical Activity$$$	832	1,21.6%; 2,12.5%; 3,15.0%; 4,50.8%	777	1,21.2%; 2,10.9%; 3,13.8%;4,54.1%	179	1,17.9%; 2,15.6%; 3,8.9%; 4,57.5%	0.155
LVEF	861	57.3±0.42	810	57.8±0.42	186	56.2±0.92	0.312
History							
Previous Myocardial	888	286 (32.2%)	836	231 (27.6%)	185	52 (28.1%)	0.101
Hypertension $	887	471 (53.1%)	835	424 (50.8%)	185	104 (56.2%)	0.344
Diabetes$	893	149 (16.7%)	838	130 (15.5%)	187	33 (17.6%)	0.696
Renal Disease$	888	87 (9.8%)	836	79 (9.4%)	184	24 (13.0%)	0.330
Alcohol (Non-Drinkers)$	896	221 (24.6%)	840	229 (27.2%)	187	44 (23.5%)	0.635
Plasma Creatinine$	865	94.2 (92.6-95.8)	818	94.4 (92.7-96.2)	187	93.5 (90.1-97.1)	0.388
BNP (pmol/l) $$	890	17.4 (16.4-18.5)	833	16.9 (15.9-18.0)	187	15.9 (14.0-18.1)	0.470
NT-proBNP (pmol/l) $$	890	76.4 (70.9-82.3)	833	77.6(72.3-83.3)	187	72.9 (62.8-84.6)	0.771
sFlt-1 (pg/mL)	249	108 (102-114)	189	104 (97.2-110)	55	109 (97.8-122)	0.554
VEGF-A (pg/mL)	276	35.9 (33.4-38.7)	215	36.3 (33.4-39.5)	58	40.9 (34.6-48.3)	0.352
Discharge Medications							
ACE inhibitor $	896	494 (55.1%)	840	488 (58.1%)	187	114 (61.0%)	0.369
β-blocker $	896	770 (85.9%)	840	744 (88.6%)	187	163 (87.2%)	0.393
Diuretic$	896	241 (26.9%)	840	233 (27.7%)	187	53 (28.3%)	0.801
Statin$	896	790 (88.1%)	840	742 (88.3%)	187	171 (91.4%)	0.539
Clopidogrel	896	458 (51.1%)	840	461 (54.8%)	187	99 (52.9%)	0.425
Amiodarone	896	40 (4.5%)	840	49 (5.8%)	187	7 (3.7%)	0.434
Angiographic measures							
Rentrop score	253	0.50±0.05	269	0.47±0.05	55	0.47±0.11	0.108
Brandt score	451	3.21 ± 0.14	463	3.42±0.15	101	3.67±0.33	0.499
Vessel Disease	458	2.01±0.04	469	2.02±0.04	101	2.10±0.09	0.723
Median Follow-Up$$$$	918	4.93 (0.14-9.45)	870	5.05 (0.13-9.48)	193	5.25 (0.11-9.49)	

$Means (SEM) or occurrence (percentage); $$Geometric mean (95% confidence interval) & adjusted for age and time to plasma sampling; $$$$ Median (range).

$$$Score of 1=sedentary, 2=<30 minutes activity on >2 days/week, 3=≥30 minutes on 2 days/week, 4= ≥30 minutes on ≥3 days/week.
